# High-Pressure Homogenization Pretreatment before Enzymolysis of Soy Protein Isolate: the Effect of Pressure Level on Aggregation and Structural Conformations of the Protein

**DOI:** 10.3390/molecules23071775

**Published:** 2018-07-19

**Authors:** Fei Zhao, Daofang Zhang, Xiangyang Li, Haizhou Dong

**Affiliations:** College of Food Science and Engineering, Shandong Agricultural University, Taian 271018, China; feizhaozhaofei@126.com (F.Z.); zhangdf0115@163.com (D.Z.)

**Keywords:** soybean protein isolates, high-pressure homogenization, antioxidant activity, static and dynamic light scattering, structure

## Abstract

The high-pressure homogenization (HPH) treatment of soybean protein isolate (SPI) before enzymatic hydrolysis using bromelain was investigated. Homogenization pressure and cycle effects were evaluated on the enzymatic degree of hydrolysis and the antioxidant activity of the hydrolysates generated. The antioxidant activity of SPI hydrolysates was analyzed by 1,1-dipheny-2-picrylhydrazyl (DPPH). The sizes and structures of the SPI-soluble aggregate after HPH treatment were analyzed using dynamic and static laser light scattering. The changes in the secondary structure, as measured by Fourier transform infrared spectroscopy (FTIR) and the macromorphology of SPI, were measured by scanning electron microscope (SEM). These results suggested that the HPH treatment (66.65%) could increase the antioxidant activities of the SPI hydrolysates compared with the control (54.18%). SPI hydrolysates treated at 20 MPa for four cycles obtained higher DPPH radical-scavenging activity than other samples. The control was predicted to be a hard sphere, and SPI treatment at 10 MPa was speculated to be Gaussian coil, polydisperse, and then the high-pressure treated SPI became a hollow sphere. Changes in the secondary structures showed protein aggregate formation and rearrangements. The image of SPI varied from a globular to a clump structure, as observed by the SEM. In conclusion, combining HPH treatment and enzymolysis could be an effective way to improve the antioxidant activity of the SPI.

## 1. Introduction

Soybean protein has been widely applied in nutritional and functional food ingredients due to its nutritional value and low price. The main components of SPI are glycinin (11S protein) and β-conglycinin (7S protein). At room temperature, glycinin forms hexameric complexes (11S) at pH 7.6, while it is mainly present in trimeric complexes (7S) at pH 3.8. Compared with β-conglycinin, native glycinin has a globular conformation and a low molecular weight, which cause poor functional properties in proteins [1].

Chemical, physical, and enzymatic modifications have been carried out to improve the functional properties of proteins. Generally, single-modification treatment has no significant efficiency. Hence, most studies concentrate on the combination of different modification methods to improve the functional properties of proteins. For instance, it has been reported that chymotrypsin hydrolysis combined with heat treatment could improve the gelling properties of whey proteins [2]. The combination of limited proteolysis with pepsin and/or high-pressure homogenization under acid conditions are a good choice to be used for the improvement of protein functional properties [1]. Many studies have shown that protein hydrolysates of plant and animal origin have significant antioxidant activity. The hydrolysates of SPI have been intensively studied and shown to possess antioxidant activities [1,3].

High-pressure homogenization (HPH), as a gentle technology, has been extensively used in the food processing industry or laboratory to modify food physicochemical properties. During the high-pressure homogenization process, a fluid ingredient is subjected to high pressures within milli-seconds. During the HPH treatment, fluids are subjected to impingement against static surfaces, cavitation, shear stress, and high turbulence. Thus, high mechanical stress is applied to suspended particles and macromolecules in a fluid, and they become deformed, twisted, and even disrupted [4]. Reports showed that high-pressure homogenization could affect the secondary structures of most globular proteins [5]. Luo et al. reported that there was a remarkable improvement on the functional properties of glycinin by using limited alcalase proteolysis combined with high-pressure homogenization [6]. Potential and current applications of HPH treatment include the functional properties of peanut proteins [7]; flaxseed oil-whey protein isolate emulsions [8]; the inactivation of enzymes in milk [9]; carotenoid transfer to oil [10]; inulin gelling [11]; and the formation of double emulsions in skim milk [12].

In this paper, we investigated the effects of limited proteolysis by bromelain and high-pressure homogenization on the degree of hydrolysis and antioxidant activities of the SPI hydrolysates. Moreover, the effect of pressure level on aggregation and structural conformations of SPI was studied by dynamic and static light scattering, FTIR and SEM. We hope to provide a practical and environmentally friendly way of improving the properties of SPI.

## 2. Materials and Methods

### 2.1. Materials

Soybean protein isolate (protein content 80.31%) was provided by Shandong Wonderful Industrial Group Co. Ltd. (Dongying, Shandong Province, China). The hydrolysis of SPI was performed using food-grade bromelain (E.C. 3.4.22.32, Guangxi Pangbo Biological Engineering Co. Ltd., Nanning, Guangxi Province, China), and the enzyme activity was 500,000 U/g.

### 2.2. Different Pretreatments of SPI Suspensions

SPI suspension (5%, *w*/*v*) was prepared as a control sample by mixing 5 g of soybean protein isolate flour with 100 mL of distilled water and then equilibrated for 30 min at room temperature. The suspension was then treated by HPH (high-pressure homogenizer, SCIENTZ-150, Ningbo Scientz Biotechnology Co. Ltd., Ningbo, Zhejiang Province, China) with cooling circulation at 30 MPa for three cycles. Thermally-treated SPI suspension (heating at 90 °C for 10 min) was also prepared for comparison. Bromelain was added in a 1:20 enzyme/substrate ratio. The mixture of protein and enzyme was incubated at 55 °C to start the enzymatic hydrolysis reaction. In this study, pH 7.0 was maintained by the addition of 1 M NaOH. After 3 h of enzymatic reaction, enzymatic hydrolysis was stopped by heating the mixture for 10 min in a 100 °C water bath. The supernatant phase was collected and centrifuged at 4000× *g* for 30 min, then freeze-dried into dried soy protein peptide for further analysis.

### 2.3. Preparation of SPI Suspensions with HPH

The HPH process was performed between 10 MPa and 90 MPa using a high-pressure homogenizer. The HPH process was performed at homogenization pressures of 10, 20, 30, 40, 50, 60, 70, 80, and 90 MPa with three cycles. SPI suspensions were subjected to HPH treatment at 30 MPa for different cycles (from one to eight cycles). The proteolysis of all samples was performed for 3 h.

### 2.4. Determination of the Degree of Hydrolysis (DH)

The DH was determined to characterize the hydrolysates by the formaldehyde titration method [13]. The percent DH was calculated according to the following equation:

(1) $$\mathrm{DH}=\frac{1000\times 0.05\times (V_{1}-V_{0})}{C\times V\times h_{\mathrm{hot}}}\times 100\%$$

where C is the concentration of the protein sample (mg/mL), V is the volume of the hydrolysates by the formaldehyde titration method, 0.05 is the molarity of sodium hydroxide (mol/L), and h_tot_ is the total number of peptide bonds in the protein substrate (mmol/g protein), while h_tot_ is calculated to be 7.75 mmol/g for SPI.

### 2.5. Determination of Antioxidant Activity of SPI Hydrolysates

The antioxidant activity of SPI hydrolysates was analyzed by 1,1-dipheny-2-picrylhydrazyl (DPPH), and the DPPH radical-scavenging activity of the SPI hydrolysates was determined by the method of Yama et al. (1998) with slight modifications [14]. Freeze-dried hydrolysate samples were dissolved in distilled water to obtain 5.0 mg/mL, respectively. An aliquot (2 mL) of the sample solution was mixed with 2 mL of 20 μM ethanolic DPPH solution. The mixture was allowed to stand for 30 min at 30 °C. The absorbance was recorded at 517 nm using a spectrophotometer. The DPPH scavenging activity of the SPI hydrolysates was calculated as follows:

(2) $$\mathrm{DPPH}\;\mathrm{scavenging}\;\mathrm{activity}\;(\%)=(1-\frac{A_{i}-A_{j}}{A_{0}})\times 100\%$$

where A_i_ is the absorbance of DPPH with the SPI hydrolysate, A_j_ is the absorbance of ethanol with the SPI hydrolysate, and A_0_ is the absorbance of DPPH with distilled water.

### 2.6. Light Scattering

#### 2.6.1. Apparatus of Light Scattering

The dynamical behavior of SPI solutions treated by HPH treatment was determined by dynamic and static light scattering using a BI-200SM dynamic laser scattering system (Brookhaven Instruments, Holtsville, NY, USA) equipped with a helium-neon laser source. The 35 mW helium neon laser (Melles Criot Laser Group, Carlsbad, CA, USA) with a wavelength of 633 nm was focused on a precision cylindrical cell (quartz, diameter: 25 mm) containing a sample solution. The samples prepared for DLS and SLS were prepared as a solution of polymer with deionized water. Prior to making the measurements, all the samples were centrifuged at 10,000 rpm for 5 min using a TG1650-WS Minispin (Shanghai, China) centrifuge. The samples were also filtered using 0.45 μm Millipore filters (Jinteng experimental equipment co. Ltd., Tianjin, China) in order to remove dust. The light scattering instrument included a precision goniometer, a photomultiplier, and a BI-200SM digital autocorrelator. All the light scattering measurements were carried out at 25 °C and controlled within 0.01 °C by a water circulating apparatus. Light scattering measurements were measured in the angular range of 30–120° for static measurements and 30–90° for dynamic measurements. Toluene was used as a reference with the Rayleigh ratio of 1.398 × 10^5^ cm^−1^ in the static light scattering measurements. The refractive index increment, dn/dc, was determined to be 0.185 mL/g for SPI in aqueous solution.

#### 2.6.2. Dynamic Light Scattering

For dynamic light scattering (DLS), the line-width distribution G (Г) can be converted to the translational diffusion coefficient distribution G (D) and to the hydrodynamic radius (R_h_) using the Stokes-Einstein equation:

R_h_ = kT/6πηD. (3)

where T is the absolute temperature, k is the Boltzmann constant, and η is the solvent viscosity. 

#### 2.6.3. Static Light Scattering

Static light scattering allows the determination of weight-average molecular weight (M_w_), radius of gyration (R_g_), and the second virial coefficient (A_2_) using the following equation:

(4) $$\mathrm{Kc}/R_{\theta }=1/M_{w}+1/(3R_{g}^{2}/M_{w})q^{2}+2A_{2c}$$

where K, c, and R_θ_ are the optical contrast factor, the concentration, and the Rayleigh ratio (scattering intensity), respectively. The q value of the scattering vector is defined as:

q = (4π/λ)sinθ/2(5)

With λ = λ_0_/n_0_, the wavelength of the light in a medium of refractive index is n_0_, and λ_0_ is the wavelength in a vacuum. The optical contrast factor K is defined by:

(6) $$K=2\pi^{2}n_{0}^{2}{\left(\mathrm{dn}/\mathrm{dc}\right)}^{2}/(N_{0}\lambda_{0}^{4})$$

where dn/dc is the refractive index increment of the solution and N_0_ is Avogadro’s number.

The Zimm method is a graphical solution to synchronously extrapolate K_c_/R_θ_ to the zero angle and the infinite dilution [15]. Kc/R_θ_ is plotted versus kc + q^2^, and k is a freely-chosen constant, which scales the contributions from c to be roughly equal to the contributions from sin^2^(θ/2). (R_g_)^2^ and A_2_ can be calculated from the slopes by extrapolating the data to zero concentrations and zero angles. M_w_ can be calculated from the intercept of both c = 0 and θ = 0 extrapolated lines.

Both dynamic and static light scattering measurements were employed simultaneously in one optical configuration, as they provide independent information about biophysical parameters of molecules in solution. The scattering data were analyzed by the cumulated method to obtain the hydrodynamic diameter and polydispersity index, which was defined by the normalized average variance of the distribution of the diffusion coefficient. Automatic mode was used for data collection in the constrained regularization (CONTIN) and non-negative least squares (NNLS) software.

### 2.7. Fourier Transformed Infrared Spectroscopy 

The IR absorption spectrum of all samples was recorded with a FTIR spectrometer (Nicolet is5, Thermo Scientific, Waltham, MA, USA). The samples for tests were prepared as potassium bromide (KBr) pellets, and then the spectra of samples were recorded in the range of 4000–500 cm^−1^. The resolution and scan number were 4 cm^−1^ and 32/sample, respectively. The SPI suspension without any treatment was used as the control. 

### 2.8. Scanning Electron Microscopy

The scanning electron microscope (SEM) analysis of the HPH-treated SPI was determined with a Supra 55 electron microscope (ZEISS, Jena, Germany) at an accelerating voltage of 5 kV. The samples were placed on a conductive carbon tape and sputter-coated with a layer of gold prior to taking images.

### 2.9. Data Analysis

Data were presented as the means ± standard deviations (SD) of three replicate determinations. Analysis of variance (ANOVA) used to determine significant difference at *p* < 0.05 using SPSS 13.0 software (SPSS Inc., Chicago, IL, USA).

## 3. Results and Discussion

### 3.1. Effect of Pretreatment on Enzymatic Hydrolysis and DPPH Radical-Scavenging Activity of the SPI Hydrolysates 

**Figure 1:**
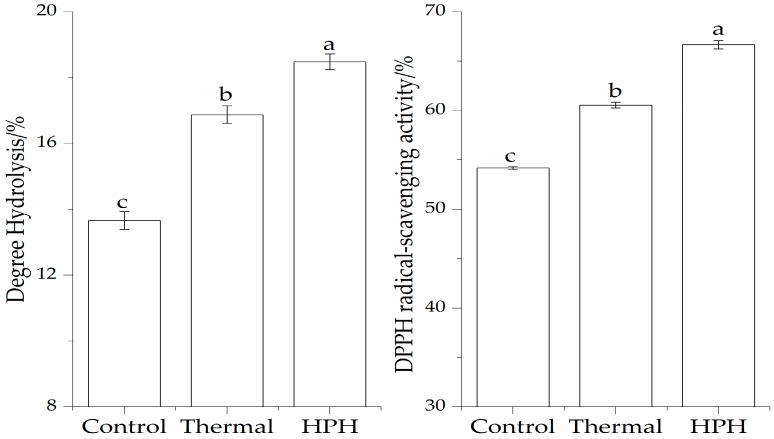
Effect of pretreatment on enzymatic hydrolysis and DPPH radical-scavenging activity of the SPI hydrolysates. Values with different letters in the same column are significantly different according to Duncan’s multiple range test (*p* < 0.05).

The enzymatic hydrolysates of the SPI substrate after two pretreatments are shown in Figure 1. The high DH of SPI obtained by thermal and HPH treatments were 16.87% and 18.48%, respectively. In contrast, without pretreatment, the DH of the SPI was only 13.66%. It can be seen that there was a significant increase (*p* < 0.05) in the DH values of the two pretreated SPI hydrolysates. These results clearly indicate that the proteolysis of SPI could be enhanced by pretreatment.

SPI after pretreatment obtained higher DPPH radical-scavenging activity than that of the control (54.18%). As observed, the DPPH radical-scavenging activity of hydrolysates obtained by thermal treatment (DH 16.87%) and HPH treatment (DH 18.48%) were 60.54% and 66.65%, respectively, which showed that the HPH pretreatment significantly (*p* < 0.05) improved the DPPH radical-scavenging activity of SPI hydrolysates. A pretreatment of the protein is an important strategy. This finding agreed with those of several previous studies [1,7].

#### 3.1.1. Effect of Pressure on Enzymatic Hydrolysis and DPPH Radical-Scavenging Activity of the SPI Hydrolysates

**Figure 2:**
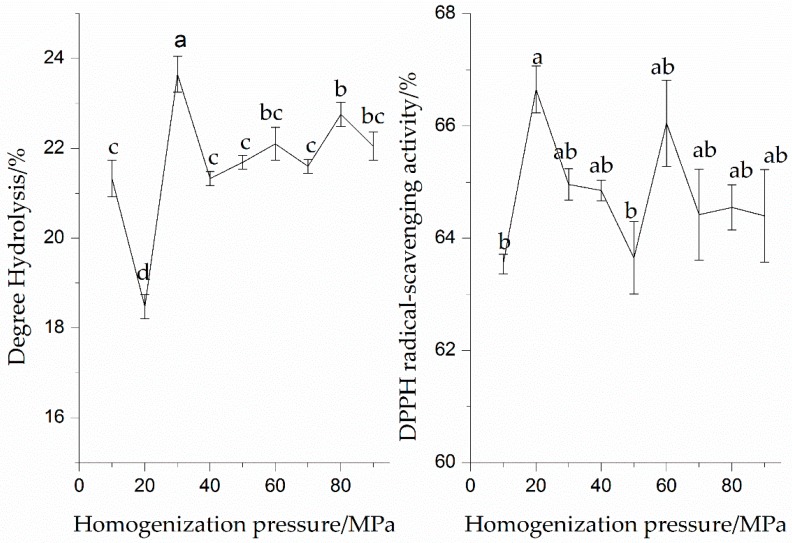
Effect of homogenization pressures on enzymatic hydrolysis and DPPH radical-scavenging activity of the SPI hydrolysates. Values with different letters in the same column are significantly different according to Duncan’s multiple range test (*p* < 0.05).

The enzymatic hydrolysates of SPI after HPH treatments under different levels of pressure are shown in Figure 2. It can also be seen that the DH value of the protein solutions increased significantly (*p* < 0.05) as the pressure of HPH treatment increased, while the DH value of SPI after HPH treatment at 30 MPa was higher than that at other pressures, which indicated that the HPH treatment at 30 MPa enhanced the enzymatic hydrolysis of SPI. Dong et al. (2009) found that the peanut protein isolate after the HPH treatment at 40 or 80 MPa exhibited a higher hydrolysis compared with that after the HPH treatment at 0.1 MPa [7]. This observation revealed that HPH treatment increases the susceptibility of enzyme cleavage sites of SPI, as well as strengthens the binding to the enzyme. The result was concerned with the denaturation, dissociation, or unfolding of SPI into monomeric proteins when the protease was present, allowing an efficient binding of the enzyme and substrate.

The effect of pressure of HPH treatment on the DPPH radical-scavenging activity of the SPI hydrolysates was investigated. The SPI hydrolysates exhibited a different scavenging activity against DPPH radicals. The DPPH radical-scavenging activity (66.65%) of the SPI hydrolysates at 20 MPa was higher than that of other treatments. Dong et al. (2009) also described that the maximum DPPH radical-scavenging activity (34.86%) of the peanut protein isolate hydrolysates was obtained when the peanut protein isolate was treated by HPH at 40 MPa [7]. García-Risco et al. (2002) found that the HPH treatment at 20 MPa could increase proteolysis of skimmed milk protein [16]. Elena et al. (2006) reported that the highest degree of milk whey proteins hydrolysis for chymotrypsin and trypsin were found at 100 and 200 MPa, respectively [17]. The results showed that the DPPH radical-scavenging activity of the SPI hydrolysates depended mainly on the proper HPH treatment level. Combining HPH treatment with enzymatic hydrolysis might be beneficial to obtain highly active antioxidant peptides and contribute to the electron-donating ability of the SPI hydrolysate. We found that the bioactive peptides generated in the hydrolysates were dependent on pressure.

#### 3.1.2. Effect of Homogenization Cycles on Enzymatic Hydrolysis and DPPH Radical-Scavenging Activity of the SPI Hydrolysates

**Figure 3:**
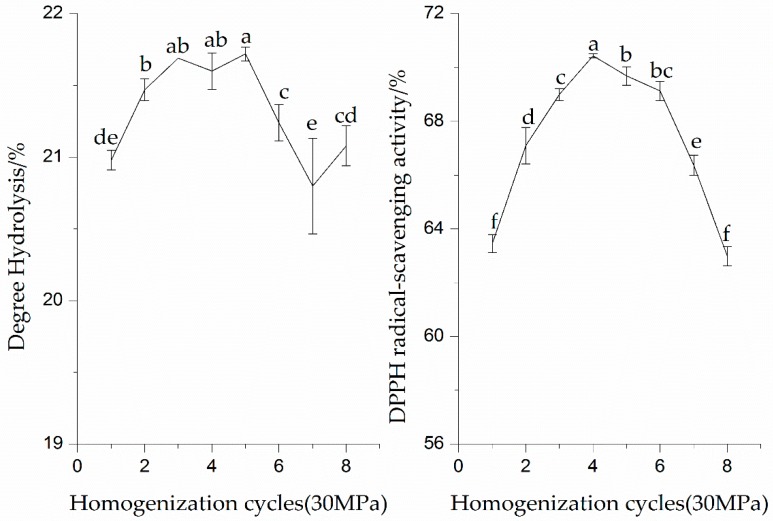
Effect of homogenization (30 MPa) cycles on enzymatic hydrolysis and DPPH radical-scavenging activity of the SPI hydrolysates. Values with different letters in the same column are significantly different according to Duncan’s multiple range test (*p* < 0.05).

As shown in Figure 3, the DH values of the SPI hydrolysates showed a significant (*p* < 0.05) increase after multiple homogenization cycles. The DH values of the SPI hydrolysates first increased to the maximum value and then decreased with increasing homogenization cycles. The SPI hydrolysates had the highest DH at five cycles of HPH treatment. This fact may be due to the protein–protein interaction changed by the HPH treatment. The susceptibility of enzyme cleavage sites of SPI was also decreased. The proteolysis of SPI could be affected by pretreatment parameters (pressure or cycle). 

As can be seen from results in Figure 3, there was a significant increase (*p* < 0.05) in the DPPH radical-scavenging activity of all proteins with the increase in the homogenization cycles. The higher DPPH radical-scavenging activity (70.44%) of the SPI was obtained when the SPI endured HPH treatment for four cycles. Furthermore, after five cycles the DPPH radical-scavenging activity of all the proteins’ hydrolysates were not further increased. The reduction in the DPPH radical-scavenging activity of the proteins may be due to the irreversible degradation of SPI induced by mechanical forces of HPH treatment. Song et al. (2013) showed that many proteins tended to unfold and particle sizes sharply decreased with the increase in the number of homogenization cycles [18]. These results revealed that the DPPH radical-scavenging activity of the SPI hydrolysates depended mainly on the proper HPH treatment cycles.

### 3.2. Light Scattering

The light scattering technique is a method useful for determining the size and structure of large aggregates, including static and dynamic light scattering. Static light scattering (SLS) relies on spectrally-resolved detection of scattered and/or angle-resolved light and is based on a fairly complex optical device. Some molecular parameters of all components in solution obtained from SLS include: weight-average molecular weight (M_w_), the second virial coefficient (A_2_), and the z-average mean radius of gyration (R_g_). Dynamic light scattering (DLS) determines the strength fluctuation with time by the measurement of a time-dependent autocorrelation function. Dynamic and static light scattering has enabled great advances in characterizing protein aggregation behavior. This technique has been successfully applied to investigate specific mechanical energy-induced aggregates of SPI, mucin, and sodium alginate/myoglobin aqueous solutions [19,20,21]. Molecular characteristics of SPI samples in aqueous solution are shown in Table 1.

#### 3.2.1. Static Light Scattering Analysis

SLS measurement was determined on the SPI solutions after continuous filtration treatment. Values of M_w_, A_2_, and R_g_ are averages (with standard deviations) obtained from two different Zimm plots (two sets of samples). Moreover, Mw values are averages obtained by extrapolating the data to both zero concentration and zero angle [22]. The M_w_, R_g_, and A_2_ of all samples are summarized in Table 1. On account of the feature of these samples, it was likely that the HPH treatment could cause the change of the SPI particle’s size. A_2_ is a measure of the interaction between the solvent and the polymer [23,24]. After HPH treatment, the A_2_ values of the SPI solutions were positive and small, while the control was negative. This observation revealed that the dissolubility of SPI solution after HPH treatment was improved. As can be seen from results in Table 1, there was a significant increase (*p* < 0.05) in the M_w_ and R_g_ of all proteins with the different homogenization pressures. Table 1 shows that HPH treatment at 10 MPa remarkably increases the M_w_ of SPI from 2.89 × 10^6^ (control) to 5.9 × 10^7^ g/mol, and the M_w_ of SPI treated by HPH treatment at 30 MPa was reduced to 2.44 × 10^7^ g/mol. This decrease in protein M_w_ was suggested to be due to the disruption of the protein aggregate caused by changes in electrostatic and hydrophobic interactions, induced by the high shear forces originating from the cavitation and high turbulence of HPH treatment. R_g_ denotes the mean square radius of the scattering solute. The R_g_ change of SPI with the pressure was similar to that of M_w_. Although the estimated R_g_ of the SPI solution at 10 MPa was about three-fold higher than the control, the M_w_ of the former was just much higher than that of the latter. The M_w_ of the SPI solution at 20 MPa was slightly lower than that at 10 MPa, while the R_g_ of the former was just half of the latter. With the increasing of homogeneous pressure, the M_w_ and R_g_ of the SPI had slight changes compared to that of 30 MPa. The data suggests that SPI aggregates are found after HPH treatment. The initial aggregates with large M_w_ seemed to be formed at low pressure levels (e.g., 10 MPa), or in the initial process at higher pressures. The initially-formed aggregates at high pressure levels would be further extended, and then dissociated into smaller aggregates. The macromolecule’s substances in the valve gap outlet endured cavitation phenomena, impacts, and turbulence which could benefit particle nucleation aggregation and aggregate disruption. Generally speaking, the HPH technique might induce changes in structure and group bonding pattern, which produces different molecular weights of protein aggregations. This result explained that the proteolysis of SPI could be enhanced by HPH treatment. 

#### 3.2.2. Dynamic Light Scattering Analysis

The SPI aggregate solutions were further described by dynamic light scattering. DLS measurements were carried out on a set of concentrations (typically 0.03–0.2 mg/mL) and in the angular range of 30–90° to consider possible inter-particle interference on scattered light. A minimal angular dependence was observed for solutions in the concentration range studied. For example, a cluster of DLS data for 0.2 mg/mL SPI after HPH treatment is shown in Table 1. The autocorrelation function appeared to decay slightly faster as the detecting angle increased. Similarly, the effects of the concentration on the DLS data were also noticeably small; the auto-correlation function decayed faster as the concentration decreased [25]. The angular dependence of hydrodynamic radius (R_h_) obtained from dynamic measurement is shown in Table 1. R_h_ is the particle’s hydrodynamic radius. By extrapolating to the zero angle, the R_h_ of the native SPI was found to be 122.97 nm, the R_h_ of the HPH-10MPa treated SPI increased to 155.67 nm, while the R_h_ decreased with the increase of the homogenization pressure. The same trend was seen in the corresponding R_g_ of these data, as shown in Table 1. This indicated that protein denatured, dissociated, or unfolded after HPH treatment, and then formed aggregates in the aqueous solution.

#### 3.2.3. The Conformation of SPI Aggregates

As static and hydrodynamic dimensions typically change with the shape of the macromolecules, it may offer information on the spatial structure of the giant molecules to combine the two measurements. The structure parameter ρ is the ratio of R_g_ obtained from SLS to the R_h_ from DLS, and is an important parameter for characterizing polymers and colloids. It depends on the polydispersity, conformation, and chain architecture, but not on the molar mass [26]. In general, the value of ρ increases with decreasing branching density, but an increase in polydispersity neutralizes the impact of branching [27]. The ρ values of the control was 0.78, which is predicted for a hard sphere, but a dramatically higher value of ρ = 1.99 was obtained for SPI-treated HPH at 10 MPa, then the higher-pressure treatment SPI (e.g., 40 MPa) with a lower value ρ (1.05). There have been several studies about the space structure of the SPI water solution induced by HPH treatment. The results suggested that the structure of the SPI solution without HPH treatment showed a hard sphere, while those subjected to HPH treatment at low pressure became Gaussian coils, poly-disperse, or became hollow spheres after being subjected to higher pressure. The spatial structure of the SPI was altered under the HPH treatment, and the globular structure of SPI may be unfolded and depolymerized, then proteins would further be associated into aggregates via hydrophobic interaction to form micelles with relatively low homogenization pressure (e.g., homogenization of SPI at <40 MPa), while the SPI micelles changed into small aggregations after HPH treatment at 40 MPa or higher pressure. This finding denoted that the presence of protein-protein and protein-solvent interaction formed differently structured aggregates at different pressure levels of HPH treatment. After HPH treatment, SPI led to protein flexibility enhancement and improved protein arrangement in a liquid solution. Then, the HPH treatment could make proteolytic enzymes sensitive to SPI and was easy to obtain high antioxidant activity of the SPI hydrolysates. The HPH treatment allowed infiltration of water into the hydrophobic zone interior of the protein substrate. Therefore, the hydration patterns of the protein would markedly influence the structural dynamic properties under the condition of high pressure, and the stability of the protein structure is primarily affected by its conformational flexibility of protein chains to compensate for losses of non-covalent bonds due to the reset of water molecules [28]. The HPH treatment can change the structure of proteins. Then hydrolysates of the SPI solution after HPH pretreatment generated higher antioxidant activity in comparison with thermal treatment. 

### 3.3. Microscopic Appearance and Structural Analysis of SPI

**Figure 4:**
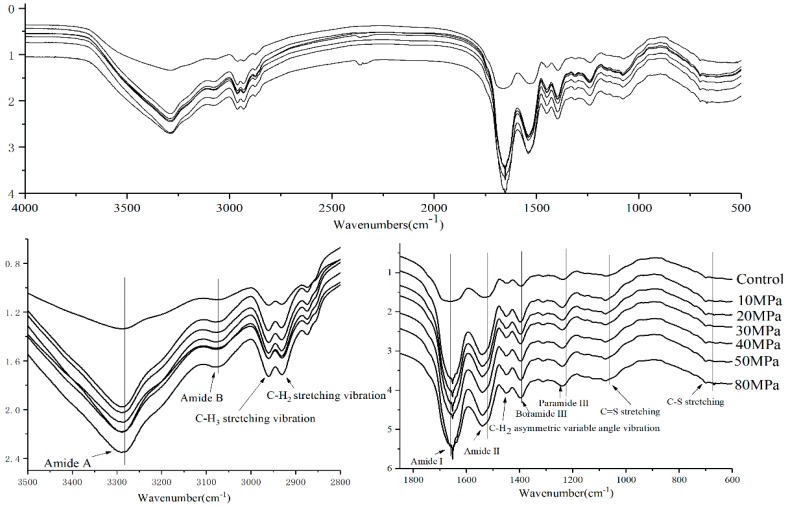
FTIR spectra of SPI after HPH treatment at different pressure levels from 4000 to 500 cm^−1^.

The secondary structures of the samples were determined by FTIR to illuminate the effect on modifying the structure of the proteins. Figure 4 describes the changes in the FTIR spectra of SPI according to characteristic shifts in some band frequencies. It was observed that the absorption bands of SPI with HPH treatment were obviously different from those of untreated SPI, especially in the Amide I (1700–1600 cm^−1^), Amide II (1560–1520 cm^−1^), and Amide III (1240–1430 cm^−1^) bands. The Amide I band is primarily caused by the C=O stretching vibration of the protein group, the Amide II band arises mainly from the C-N stretching with N-H bending vibrations of the protein backbone, and the Amide III is generated by C-N stretching [29]. The effect of high pressure can be systematically investigated. From Figure 4, it was observed that the HPH treatment increased the Amide I and II band intensities with a sharpened peak shape. Generally, the HPH-treated SPI had higher absorbance than untreated SPI between 4000 and 400 cm^−1^. HPH treatment led to a significant bathochromic shift (about 2 cm^−1^) of the Amide I band peaks of SPI, and hypsochromic shifts of Amide II (about 6 cm^−1^), III, A (about 3 cm^−1^) and Amide B (about 2 cm^−1^) band spectra of SPI. This is indirect evidence for HPH-induced changes of the protein’s structure. A similar result was gained by Tang et al. who worked on aggregation and structural properties of soy protein isolate treated with high-pressure [5].

The Amide I band is composed of some bands corresponding to the diverse secondary structure of proteins. The secondary derivative spectra of SPI were fitted by the Gaussian peak to show the characteristic peaks, e.g., the α-helix (1650–1658 cm^−1^), β-sheet (1610–1640 cm^−1^), β-turn (1660–1670 cm^−1^), and random coil (1640–1650 cm^−1^) [30]. Then the secondary structure contents of SPI are determined by calculating the peak areas. Table 2 shows the results of the contents. Compared with the untreated-SPI, the α-helix, β-sheet, and random coil contents of SPI were reduced significantly by HPH treatments, while their β-turn content was increased. The pressure significantly (*p* < 0.05) decreased the portion of α-helix, β-sheet, and random coil contents of SPI while increasing that of the β-turn. Compared to the control, α-helix content of 80 MPa-treated SPI decreased by 24.34%, β-sheet and random coil contents of 50 MPa treated SPI decreased by 17.11% and 16.12%, and that of β-turn increased by 29.76%, respectively. This observation indicated that the α-helix, β-sheet, and random coil were transformed to the β-turn. Wei et al. (2018) reported a significant decrease in α-helix and β-sheet, and an increase in random coil and β-turn of the protein after high hydrostatic pressure treatment [31]. The changes of pressure-induced glycinin preparations indicated partial loosening in α-helix and β-sheet structures in glycinin samples of 60% (*w*/*w*) solids [32]. The diversities between our results and those views were caused by different HPH treatment parameters, such as pressures and concentration. The Amide I band is the most sensitive IR spectral region to predict the secondary structural components of proteins and is mainly attributed to C=O stretching vibrations (about 80%) with some in-plane N-H bending and C-H stretching modes. In particular, the C=O stretching vibrations of proteins primarily depend on the different secondary structures and intra- or intermolecular effects, including hydrogen bonding pattern and molecular geometry [33]. This result indicated that the secondary structure of SPI changed largely under HPH treatment.

By detecting changes in spectra at 1449 cm^−1^ (C-H_3_ asymmetric variable angle vibration), as well as at 2932 cm^−1^ (C-H_2_ stretching vibration), at 2961 cm^−1^ (C-H_3_ stretching vibration) (Figure 4) [29], the microenvironment of aliphatic amino acid residues was obtained. No significant changes by HPH treatment were observed for the C-H_2_ stretching vibration and C-H_3_ stretching vibration band intensity, and 1449 cm^−1^ (C-H_3_ asymmetric variable angle vibration) band spectrum showed a hypsochromic shift. From Figure 4, it is observed that HPH treatment causes an increase of the spectral absorption at 730–600 cm^−1^ (C-S stretching) and 1153 cm^−1^ (C=S stretching), suggesting changes of reactive groups (such as sulfhydryl groups and hydrophobic groups). Clearly, the hypsochromic shift of the FTIR spectrum peak can be explained by the HPH effect on SPI. HPH pretreatment may expose the chromophore and auxochrome groups in protein. The former are C=C, C=O, N=N, N=O, and -COOR, the latter are -OH, -NH_2_, and -SH. The secondary structure of protein would unfold and disassociate during HPH processing, while the original structure remains unchanged [34].

In theory, the substrate pretreatment should make the protein denature and expose new active sites on this protein, like with HPH treatment. The changes in the secondary structure suggested that HPH pretreatment make the exposure of more hydrophobic groups and regions inside the protein and the unfolded protein structure being more beneficial to enzymatic hydrolysis. Furthermore, the antioxidant activity of SPI hydrolysate treated with HPH treatment was higher than that without HPH treatment.

### 3.4. SPI Film Macromorphology

**Figure 5:**
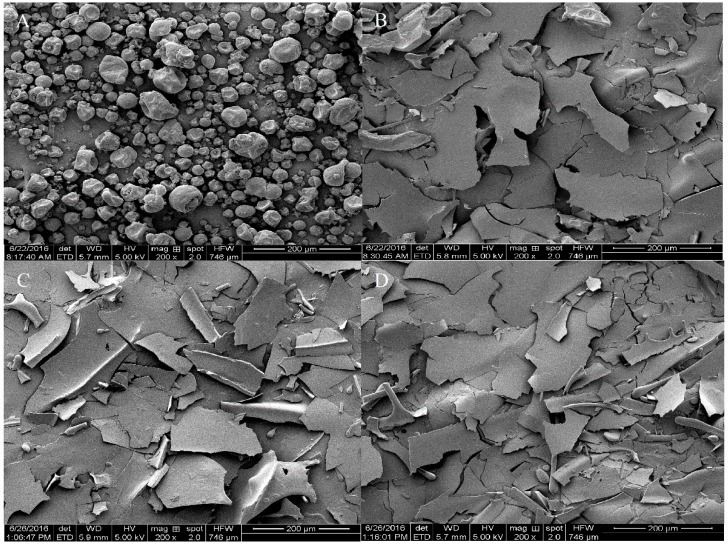
Scanning electron micrographs of SPI formed from non-HPH treated SPI (**A**); 10 MPa HPH-treated SPI (**B**); 40 MPa HPH-treated SPI (**C**); and 80 MPa HPH-treated SPI (**D**).

In order to study the effect of different HPH treatments on SPI, the macrostructure of SPI was investigated by SEM. Figure 5 revealed a set of SEM images of SPI alternatively treated at a magnification factor of 200-fold. The image of untreated SPI (Figure 5A) exhibited a wrinkled surface and spherical shape with cavities, while the spherical structure of SPI disappeared after HPH treatment. Feng Liu et al. (2016) reported native SPI powders with a capsular structure [35]. The smoother and finer structures were observed from the HPH-treated SPI images (Figure 5B–D) with the application of higher pressure. SPI after high-pressure homogenization aggregated into a larger size, and few spherical structures of the SPI were also observed from the HPH-treated SPI image (Figure 5B) [6]. In the case of SPI-40 MPa, the HPH treatment also induced SPI to form large clumps (Figure 5C). SPI-80 MPa (Figure 5D) had the most fragmented clump structure among the three HPH-treated samples. It was observed that protein isolates after HPH treatment showed clumps with different shapes and sizes. This indicates that HPH treatment can produce clump structures from the globular structure.

## 4. Conclusions

High-pressure homogenization is a non-thermal technology. In recent years, this technology has been used for improving the physicochemical characteristics of materials and products. In this paper, HPH pretreatment in combination with enzymatic hydrolysis was found to increase the antioxidant activities of the SPI hydrolysates. HPH-induced aggregation and conformational changes of soy proteins have been successfully characterized using DLS, SLS, FTIR, and SEM techniques. The results revealed that the SPI water solutions with different pressure levels of HPH treatment formed various hydrodynamic sizes and space structures of protein aggregates, as measured by DLS and SLS. The mechanical impact of HPH treatment on SPI caused the changes in the chemical and physical structure of proteins, and then led to changes of the antioxidant activities of SPI. The results in this study provide a theoretical basis for further study of protein treatment and will potentially be useful for comprehensive utilization of SPI, which can be widely applied in many fields.

## Figures and Tables

**Table 1:** Summaries of the dynamic and static light scattering data on the soluble aggregates in the SPI aqueous solutions with HPH treatment.

Sample Code	M_w_ (g/mol × 10^7^)	R_g_ (nm)	R_h_ (nm)	A_2_ (×10^-4^)	ρ (R_g_/R_h_)	Structure
Control	0.29 ± 0.17 ^e^	96.0 ± 5.4 ^e^	122.97	–3.28 ± 0.65	0.78	hard sphere
HPH-10 MPa	5.90 ± 0.22 ^a^	310.0 ± 6.4 ^a^	155.67	1.60 ± 0.41	1.99	GCP
HPH-20 MPa	4.36 ± 0.34 ^b^	161.0 ± 8.3 ^b^	140.18	0.72 ± 0.18	1.15	GCM
HPH-30 MPa	2.44 ± 0.14 ^d^	141.0 ± 6.0 ^c^	117.14	1.45 ± 0.5	1.2	GCM
HPH-40 MPa	2.24 ± 0.08 ^d^	122.2 ± 3.4 ^d^	115.94	1.38 ± 0.32	1.05	hollow sphere
HPH-60 MPa	2.89 ± 0.11 ^c^	114.3 ± 3.7 ^d^	109.11	0.74 ± 0.22	1.05	hollow sphere

Weight-average molecular weight (M_w_), z-average mean radius of gyration (R_g_), hydrodynamic radius (R_h_), the second virial coefficient (A_2_), Gaussian coil, polydisperse (good solvent) (GCP), and Gaussian coil, monodisperse (θ-solvent) (GCM). Values with different letters in the same column are significantly different according to Duncan’s multiple range test (*p* < 0.05).

**Table 2:** Effects of HPH treatment on secondary structure composition of SPI.

Pressure (MPa)	α-Helix (%)	β-Sheet (%)	β-Turn (%)	Random Coil (%)
0	20.21 ^a^	27.23 ^a^	33.33 ^c^	19.23 ^a^
10	16.99 ^d^	22.84 ^e^	43.86 ^a^	16.99 ^c^
20	17.27 ^c^	22.74 ^e^	43.78 ^a^	16.18 ^e^
30	16.43 ^e^	22.99 ^d^	43.69 ^a^	16.89 ^c^
40	16.15 ^f^	23.42 ^c^	44.04 ^a^	16.39 ^d^
50	17.82 ^b^	22.57 ^f^	43.47 ^b^	16.13 ^e^
80	15.29 ^g^	23.67 ^b^	43.25 ^b^	17.79 ^b^

Values with different letters in the same column are significantly different according to Duncan’s multiple range test (*p* < 0.05).

## References

[1] Boen Y., Ren J., Zhao M., Luo D., Gu L. (2012). Effects of limited enzymatic hydrolysis with pepsin and on the functional properties of soybean protein isolate. LWT Food Sci. Technol..

[2] Rickert D.A., Johnson L.A., Murphy P.A. (2004). Functional properties of improved glycinin and b-conglycinin fractions. J. Food Sci..

[3] Ngoh Y.-Y., Lim T.S., Gan C.-Y. (2016). Screening and identification of five peptides from pinto bean with inhibitory activities against α-amylase using phage display technique. Enzym. Microb. Technol..

[4] Floury J., Bellettre J., Legrand J., Anne D. (2004). Analysis of a new type of high pressure homogeniser: A study of the flow pattern. Chem. Eng. Sci..

[5] Tang C.-H., Ma C.-Y. (2009). Effect of high pressure treatment on aggregation and structural properties of soy protein isolate. LWT Food Sci. Technol..

[6] Luo D., Zhao Q., Zhao M., Yang B., Long X., Ren J., Zhao H. (2010). Effects of limited proteolysis and high-pressure homogenisation on structural and functional characteristics of glycinin. Food Chem..

[7] Dong X., Zhao M., Shi J., Yang B., Li J., Luo D., Jiang G., Jiang Y. (2011). Effects of combined high-pressure homogenization and enzymatic treatment on extraction yield, hydrolysis and function properties of peanut proteins. Innov. Food Sci. Emerg. Technol..

[8] Kuhn K.R., Cunha R.L. (2012). Flaxseed oil-Whey protein isolate emulsions: Effect of high pressure homogenization. J. Food Eng..

[9] Hayes M.G., Fox P.F., Kelly A.L. (2005). Potential applications of high pressure homogenizationin-processing of liquid milk. J. Dairy Res..

[10] Leonard M., Agnese P., Edwin T., Musabe A.V.L., Marc H. (2015). Carotenoid transfer to oil upon high pressure homogenisation of tomato and carrot based matrices. J. Funct. Foods.

[11] Saioa A.-S., Inigo M.D.M., Juan-Carlos A. (2015). Impact of high pressure homogenisation (HPH) on inulin gelling properties, stability and development during storage. Food Hydrocoll..

[12] Thomas S.H.L., Zhou M., Zhou D., Muthupandian A., Gregory J.O.M. (2018). The formation of double emulsions in skim milk using minimal foodgrade emulsifiers—A comparison between ultrasonic and high pressure homogenisation efficiencies. J. Food Eng..

[13] Zhao X., Feng Z. (1994). Determination for degree of hydrolysis of the protein hydrolysates. Food Sci..

[14] Yamagucei T., Tasamura H., Matob A., Junji T. (1998). HPLC method for evaluation of the free radical-scavenging activity of foods by using l, l-dipheny l-2-picrylhydrazy. Biosci. Biotechnol. Biochem..

[15] Li W., Steve W., Wang C.Q., Rickey Y.Y. (2011). Studies of aggregation behaviours of cereal β-glucans in dilute aqueous solutions by light scattering: Part I., Structure effects. Food Hydrocoll..

[16] García-Risco M.R., Ramos M., López-Fandino R. (2002). Modifications in milk proteins induced by heat treatment and homogenization and their influence on susceptibility to proteolysis. Int. Dairy J..

[17] Elena P., Guadalupe P., Maria L.B., Maria I.M.-M., Rosario G. (2006). Effects of combined high pressure and enzymatic treatments on the hydrolysis and immunoreactivity of dairy whey proteins. Int. Dairy J..

[18] Song X., Zhou C., Fu F., Chen Z., Wu Q. (2013). Effect of high-pressure homogenization on particle size and film properties of soy protein isolate. Ind. Crops Prod..

[19] Fang Y., Zhang B., Wei Y., Li S. (2013). Effects of specific mechanical energy on soy protein aggregation during extrusion process studied by size exclusion chromatography coupled with multi-angle laser light scattering. J. Food Eng..

[20] Peter C.G., Beatrice C., Mervat S.I., Josephine C.A. (2015). Probing the interaction of nanoparticles with mucin for drug delivery applications using dynamic light scattering. Eur. J. Pharm. Biopharm..

[21] Caterina B., Ulderico W., Giovanna D.A., Cristina C., Simona R. (2015). Study of the dynamical behavior of sodium alginate/myoglobin aqueous solutions: A dynamic light scattering study. J. Mol. Liq..

[22] Enoki T.A., Henriques V.B., Lamy M.T. (2012). Light scattering on the structural characterization of DMPG vesicles along the bilayer anomalous phase transition. Chem. Phys. Lipids.

[23] Burchard W., Ross-Murphy S.B. (1994). Light scattering techniques. Physical Techniques for the Study of Food Biopolymers.

[24] Li W., Wang Q.C., Cui S.W., Huang X., Kakuda Y. (2006). Elimination of aggregates of (1→3) (1→4)-beta-d-glucan in dilute solutions for light scattering and size exclusion chromatography study. Food Hydrocoll..

[25] Sonia Z., Ingo L., Muhammad I.S. (2008). Soluble aramid containing ether linkages: Synthesis, static and dynamic light scattering studies. Chem. Phys..

[26] Kirkwood J.G., Riseman J. (1948). The intrinsic viscosities and diffusion constants of flexible macromolecules in solution. J. Chem. Phys..

[27] Wang Q., Huang X., Akihiro N., Walther B., Hallett F.R. (2005). Molecular characterisation of soybean polysaccharides: An approach by size exclusion chromatography, dynamicand static light scattering methods. Carbohydr. Res..

[28] Buckow R., Sikes A., Tume R. (2013). Effect of high pressure on physicochemical properties of meat. Crit. Rev. Food Sci. Nutr..

[29] Weng S., Xu Y. (2016). Fourier Transform Infrared Spectroscopy.

[30] Wang C., Jiang L., Wei D., Li Y., Sui X., Wang Z., Li D. (2018). Effect of Secondary Structure determined by FTIR Spectra on Surface Hydrophobicity of Soybean Protein Isolate. Procedia Eng..

[31] Wei J., Zhang Z., Cai Q., Peng B. (2018). Effects of high hydrostatic pressure on structural and physical properties of nisin-SPI film. Int. J. Biol. Macromol..

[32] Sobhan S., Anna B., Nitin M., Stefan K. (2016). Structural modification in condensed soy glycinin systems following application of high pressure. Food Hydrocoll..

[33] Kong J., Yu S. (2007). Fourier transform infrared spectroscopic analysis of protein secondary structures. Acta Biochim. Biophys. Sin..

[34] Tabilo-Munizaga G., Gordon T.A., Villalobos-Carvajal R., Moreno-Osorio L., Salazar F.N., Perez-Won M., Sergio A. (2014). Effects of high hydrostatic pressure (HHP) on the protein structure and thermal stability of Sauvignon blanc wine. Food Chem..

[35] Feng L., Hailiang Z., Jianbing P., Jinwen H., Hongbo L., Yanwu C., Fenghui L. (2016). Removal of copper(II) using deacetylated konjac glucomannan conjugated soy protein isolate. Int. J. Biol. Macromol..

